# A serum- and feeder-free technique of culturing human corneal epithelial stem cells on amniotic membrane

**Published:** 2009-06-30

**Authors:** Kaevalin Lekhanont, Lulin Choubtum, Roy S. Chuck, Tarinee Sa-ngiampornpanit, Varintorn Chuckpaiwong, Anun Vongthongsri

**Affiliations:** 1 Department of Ophthalmology, Ramathibodi Hospital, Mahidol University, Bangkok, Thailand; 2 Ramathibodi Research Center, Ramathibodi Hospital, Mahidol University, Bangkok, Thailand; 3 Wilmer Eye Institute, Johns Hopkins University, Baltimore, MD

## Abstract

**Purpose:**

To describe a simple technique of cultivating human corneal epithelial stem cells using an Epilife® culture medium under serum- and feeder-free conditions.

**Methods:**

Cadaveric donor limbal corneal epithelial cells were cultured on denuded amniotic membranes using an explant technique that was free of serum and feeder cells in the Epilife® medium containing a growth supplement of defined composition. These cells were assessed by phase contrast microscope. The expressions of the proposed corneal epithelial stem cell markers (p63, ATP-binding cassette member 2 (ABCG2), and cytokeratin 15 and 19) and differentiation markers (cytokeratin 3, 12, connexin 43, and p75) were analyzed using reverse transcription polymerase chain reaction (RT-PCR) and immunocytochemical staining.

**Results:**

Successful cultures were obtained, resulting in a monolayer to double layer cell sheets with a cobblestone-like morphology. RT-PCR and immunocytochemistry disclosed an expression of both putative limbal stem cell (LSC) markers and differentiation-associated markers in the cultured cells. Most of the cultured corneal epithelial cells that were immunopositive for putative LSC markers were smaller, more uniform, and closer to the limbal explant than cells positively stained with differentiation-associated markers.

**Conclusions:**

A serum- and feeder-free culture system using Epilife® medium may grow human corneal epithelial equivalents, minimizing the risk of contamination during culture. The technique may also be useful for the clinical application of limbal stem cell culture.

## Introduction

The ocular surface is covered by three anatomically different epithelia, the corneal, conjunctival, and limbal epithelia. The corneal epithelium is a non-keratinized stratified squamous epithelium, which is responsible for maintaining ocular surface health and is essential for good vision. Since the corneal epithelium has a finite life span, its renewal is particularly important to support corneal structure and function. It is now known that corneal epithelial stem cells are located in the basal layer of the limbus where a corrugated, pigmented structure called the palisades of Vogt is observed in some races [1,2]. During homeostasis and following injury, these limbal epithelial stem cells (LESCs) govern the renewal of the corneal epithelium by regenerating transient-amplifying cells that migrate centripetally from the limbus into the corneal basal layer, proliferating and differentiating to replace lost corneal epithelial cells [3,4]. In addition to replenishing the corneal epithelium, the LESCs also form a barrier to prevent the encroachment of the conjunctival epithelium onto the surface of the cornea [5]. Loss or dysfunction of LESCs, which is described by the clinical entity of limbal stem cell deficiency (LSCD), results in the drying up of the source of corneal epithelial cells and the invariably inward invasion of the adjacent conjunctiva. This can lead to corneal vascularization, chronic inflammation, corneal opacification, and severe visual loss. LSCD can occur in several diseases such as Stevens Johnson syndrome, ocular cicatricial pemphigoid, chemical or thermal injuries, severe dry eye syndrome, multiple ocular surgeries, contact-lens induced ocular surface disease, and hereditary disorders. In such diseases, traditional penetrating keratoplasty has a guarded prognosis because it does not replace LESCs, which are necessary for the ongoing renewal of the corneal epithelium. Therefore, many attempts have been made to establish alternative surgical treatments for severe LSCD-associated ocular surface diseases. Therapeutic limbal transplantation, which involves the transplantation of large pieces of healthy limbus from either the fellow unaffected eye or the eye of related living or cadaveric donors, in conjunction with amniotic membrane transplantation have been developed [6-8]. However, this techniques have major disadvantages. The surgeries may fail and lack longevity if the limbal grafts contain inadequate stem cells. Limbal epithelial exhaustion of the healthy donor eye is also another concern in the case of autografts or allografts from living related donors if the amount of stem cells is too much removed from the donor eye [9,10]. Additionally, allograft transplantation carries the risk of graft rejection, requiring the concomitant aggressive systemic immunosuppression to enhance graft survival [11]. Nonetheless, the long-term success rate still remains low despite potent immunosuppressive therapy [8,12].

Moreover, the intense immunosuppression is associated with morbidity and reduction in the patient’s quality of life [13]. With the advanced knowledge of limbal stem cell (LSC) biology, recent efforts have been made to cultivate and amplify limbal stem cells ex vivo for transplantation onto a damaged cornea [14-16]. This technique overcomes the limitations related to whole limbal tissue transplantation technique. The living donor eye is less likely to develop iatrogenic LSCD because much smaller amounts of limbal tissue are removed. The possibility that the patient’s other eye can be used as the source of LSC for expansion and subsequent transplantation is also higher, avoiding the necessity of postoperative immunosuppressive therapy. Early clinical trials of the transplantation of cultivated corneal epithelial stem cells have shown encouraging results [15-23]. To date, there are a variety of different methods of cultivating limbal epithelium, which can be mainly categorized into three techniques. The first technique, which is based on the original protocol by Rheinwald and Green [24] for the expansion of human epidermal keratinocytes, involves the co-culture of the limbal epithelium with a mitotically inactivated 3T3 mouse fibroblast feeder layer. The second involves the use of the human amniotic membrane (HAM) as a growth substrate and carrier for limbal cell culture. The other cell carrier alternatives that have been used include fibrin glue [19], fibrin gel [25,26], and temperature responsive polymer [27,28]. However, HAM appears to be the most preferable carrier system because it is easily obtained and provides a strong, biodegradable, and easily manipulated carrier for cells [29]. In addition, it facilitates the growth and expansion of limbal epithelial cell without the need for 3T3 fibroblasts and maintains stem cell characteristics in ex vivo culture [30-34]. HAM is also non-immunogenic and has several unique properties that render it useful in ocular surface surgery such as inhibition of inflammation, vascularization and scar formation, and promotion of corneal epithelialization [35-37]. The third method is a combination of the first two techniques, using both a growth-arrested murine fibroblast feeder layer and HAM [16-18,38]. Among different techniques, fetal bovine serum (FBS)-supplemented culture medium is the most widely used medium in the culture process. However, the use of animal cells and FBS raises concerns about the risk of transmission of zoonotic infection or unknown pathogens.

Therefore, we modified the culture method by not using FBS and murine feeder cells to reduce the risk of contamination or disease transmission during culture. In this study, we describe a simple technique of cultivating corneal epithelial stem cells under serum- and feeder-free conditions using the Epilife® culture medium. To our knowledge, the quality of human corneal epithelial stem cells cultured in Epilife® media has not been previously reported.

## Methods

This study was approved by the institutional review board of Human and Research Ethics Committee of Mahidol University School of Medicine (Bangkok, Thailand). All experimental procedures were conducted in accordance with the Declaration of Helsinki. All human tissue was obtained with informed consent for involvement in laboratory research.

### Materials and reagents

Cell culture plasticware was purchased from BD Biosciences (Lincoln Park, NJ). Epilife® basal medium and growth supplements were from Cascade Biologics (Portland, OR). Other cell culture reagents were from Invitrogen-Gibco (Grand Island, NY). Mouse anti-human cytokeratin 3 (K3) antibody was from Sigma-Aldrich (St. Louis, MO). Goat anti-human cytokeratin 12 (K12) antibody was from Santa Cruz Biotechnology (Santa Cruz, CA). Mouse anti-human cytokeratin 15 and 19 antibody (K15, K19) was from Millipore (Billerica, MA). Mouse anti-human connexin 43, nerve growth factor (NGF) receptor (p75), ATP-binding cassette member 2 (ABCG2), and p63 antibodies were from BD Biosciences. Fluorescein isothiocyanate (FITC)-conjugated rabbit anti-mouse secondary antibody was also from BD Biosciences.

### Human donor tissues

Cadaveric human limbal tissues were obtained from the corneoscleral rings remaining from corneoscleral buttons provided for corneal transplantations by the Thai Red Cross Eye Bank (Bangkok, Thailand) within five days after harvesting. The age of donors ranged from 47 to 70 years. The details of the donors’ conditions, tissue procurement, and length of preservation were given by the eye bank. These tissues were stored in Optisol^™^-GS (Bausch and Lomb Inc., Rochester, NY) at 4 °C until processed for culture.

### Human amniotic membrane

With proper written consent, HAMs were obtained from placentas donated during elective cesarean section deliveries. The membranes were washed three times under sterile conditions with phosphate-buffered saline (PBS) containing antibiotics (0.5 mg penicillin, 0.5 mg streptomycin, and 1 mg neomycin; PSN antibiotic mixture) and an antifungal agent (2.5 mg amphotericin B) and then preserved at −80 °C in Dulbecco’s modified Eagle’s medium (DMEM) and glycerol (Gibco BRL, Rockville, MD) at the ratio of 1:1 (vol/vol) [39]. HAM was supplied as individual units measuring approximately 2×3 cm^2^ mounted on nitrocellulose paper. Before use, HAM was pretreated with 0.25% trypsin in 0.02% EDTA for 15–30 min to remove the amniotic epithelium. The denuded amniotic membrane was ready for corneal epithelial cell cultures.

### Cultivation of corneal limbal explants on denuded HAM

Corneal epithelial cells were grown from limbal explants using a modification of a previously reported culture system [17]. Briefly, the corneoscleral tissues were rinsed with phosphate buffer solution containing 100 U/ml penicillin, 50 µg/ml gentamicin, and 2.5 µg/ml amphotericin B. Under a surgical microscopy, the central cornea, excess sclera, iris, corneal endothelium, conjunctiva, and Tenon’s capsule were carefully removed. Each remaining limbal ring was then divided into 1×1 mm^2^ segments. Three pieces of the segments were placed epithelial side up at the center of acellular HAM, which was spread on the glass slide and placed in a tissue culture well. The explants were cultured in Epilife® basal medium with a growth supplement of defined composition. The growth supplement was composed of purified BSA, purified bovine transferrin, hydrocortisone, recombinant human insulin-like growth factor type-1, prostaglandin E2, and recombinant human epidermal growth factors (EDGS; Cascade Biologics). Additionally, 10 µg/ml gentamicin and 0.25 µg/ml amphotericin B were added in the culture medium. All cultures were incubated at 37 °C with a humidified atmosphere containing 5% CO_2_. The medium was changed every two days. Cultivation of the cells was continued for three weeks with direct monitoring every two to three days using a phase contrast microscope. After ending the cultures, the epithelial cells were released and separated from the underlying HAM by treating with 0.05% trypsin for 10 min at 37 °C. These cells were analyzed for the expression of proposed corneal epithelial stem cell markers and differentiation markers.

### RNA extraction and reverse transcription polymerase chain reaction

Total RNA was extracted from cultured cells using TRIzol reagent (Invitrogen Life Technologies, Carlsbad, CA) according to the manufacturer’s instructions. The RNA was quantified by its absorption at 260 nm, and the quality of RNA was checked by gel electrophoresis. The extracted RNA was stored at −80 °C ready for use in reverse transcription polymerase chain reaction (RT-PCR). The first stage of reverse transcription involved DNase treatment of RNA to eliminate the residual DNA from RNA. Briefly, the mixture of 1 µg of RNA and 1 µl of 10X DNase reaction buffer was treated with 1 µl DNase I (Amp Grade; Invitrogen Life Technogies) and made up to a volume of 10 µl with RNase-free water. The solution was incubated for 15 min at room temperature after which 1 µl of 25 mM EDTA solution was added to inactivate the DNase I. The solution was then incubated at 65 °C for 10 min. First strand cDNAs were synthesized with random hexamers using a Superscript^TM^ III First Strand cDNA Synthesis Supermix (Invitrogen life Technologies). PCR amplification of the first strand cDNAs was performed with specific primer pairs that were designed from published human gene sequences for different markers (Table 1). All PCR amplification reactions were run following a standard protocol with a housekeeping gene, glyceraldehydes-3-phosphate dehydrogenase (*GAPDH*), as internal control. In brief, samples were prepared in a 25 µl volume reaction containing 1X PCR buffer (20 mM Tris-HCL pH 8.0, 1mM dithiothreitol (DTT), 0.1 mM EDTA, 100 mM KCL), 1.5 mM MgCl_2_, and 0.2 µM of each primer. The concentration of each of the four dNTPs was 0.2 mM. cDNA template concentrations varied from 200 ng to 500 ng. The PCR mixture was initially denatured at 94 °C for 7 min followed by amplification of 35 cycles each at 94 °C for 1 min, primer specific annealing temperature (Table 1) for 1 min, and 72 °C for 1 min. After amplification, the PCR products were separated on 1.5% agarose gel in 1X Tris-boric acid-EDTA (TBE) buffer containing 0.5 µg/ml ethidium bromide. Gels were photographed and scanned.

**Table 1:** Human primer pairs used for RT-PCR.

**Primer**	**Sequence (5′-3′)**	**Size of PCR product** **(base pairs)**	**Annealing temperature (°C)**
*K3*_F	CAGAATGCCAACCTGCAGAC	569	66
*K3*_R	GAGTAGCGCTGGGAGGACT		
*K12*_F	GAGCTCCAAAGCTTCCGGGTGGGC	675	62
*K12*_R	CATTAGTTCTTCAATTTCCTGAAC		
*K15*_F	GGCCACCACCATCGACAACTC	520	70
*K15*_R	GCTGAGCTGGGACTGCAGCT		
*K19*_F	GGCAACGAGAAGCTAACCATGC	469	65
*K19*_R	TGACCTGGCCTCCCACTTGG		
*ΔNp63α*_F	GGAAAACAATGCCCAGACTC	1389	64
*ΔNp63α*_R	ATGATGAACAGCCCAACCTC		
*p75*_F	TGAGTGCTGCAAAGCCTGCAA	230	55
*p75*_R	TCTCATCCTGGTAGTAGCCGTAG		
*ABCG2*_F	AGTTCCATGGCACTGGCCATA	379	60
*ABCG2*_R	TCAGGTAGGCAATTGTGAGG		
*Connexin 43*_F	TCAAGCCTACTCAACTGCTGGAG	406	63
*Connexin 43*_R	CCCTCGCATTTTCACCTTACC		
*GAPDH*_F	GATGCCCCCATGTTCGTCATG	493	66
*GAPDH*_R	GGGTGTCGCTGTTGAAGTCAG		

Forward and reverse primer sequences with corresponding annealing temperatures were used for RT-PCR in this study.

### Immunocytochemistry

Immunocytochemical staining was performed to evaluate the expression of different molecular markers that have been proposed to identify epithelial stem cells and differentiated cells. Briefly, corneal limbal epithelial cells cultured on coverslips at 70%–80% confluence were fixed with cold methanol (for cytoplasmic and nuclear protein staining) or 4% paraformaldehyde (for membrane protein staining) for 10 min at room temperature. Cells were blocked and permeabilized with 3% BSA/0.3% Triton X-100/PBS for 30 min. Primary antibodies against K3 (1:100), K12 (1:100), K15 (1:100), K19 (1:100), connexin 43 (1:100), p75 (1:25), ABCG2 (1:100), and p63 (1:25) [40] were applied and incubated for 2 h at room temperature in a humidifier chamber followed by incubation with FITC-conjugated secondary antibody for 30 min according to the manufacturer’s protocol (BD Biosciences). After proper staining with the secondary antibody, the coverslips were inverted (cell side down) and mounted with a mounting medium (PBS:glycerol with a ratio of 1:9). The cultured cells were examined under a fluorescent microscope. Negative isotype controls were used when imaging pictures to ascertain that there was no false positive staining.

## Results

### Cultivation of corneal epithelial stem cells in Epilife® culture medium

**Figure 1:**
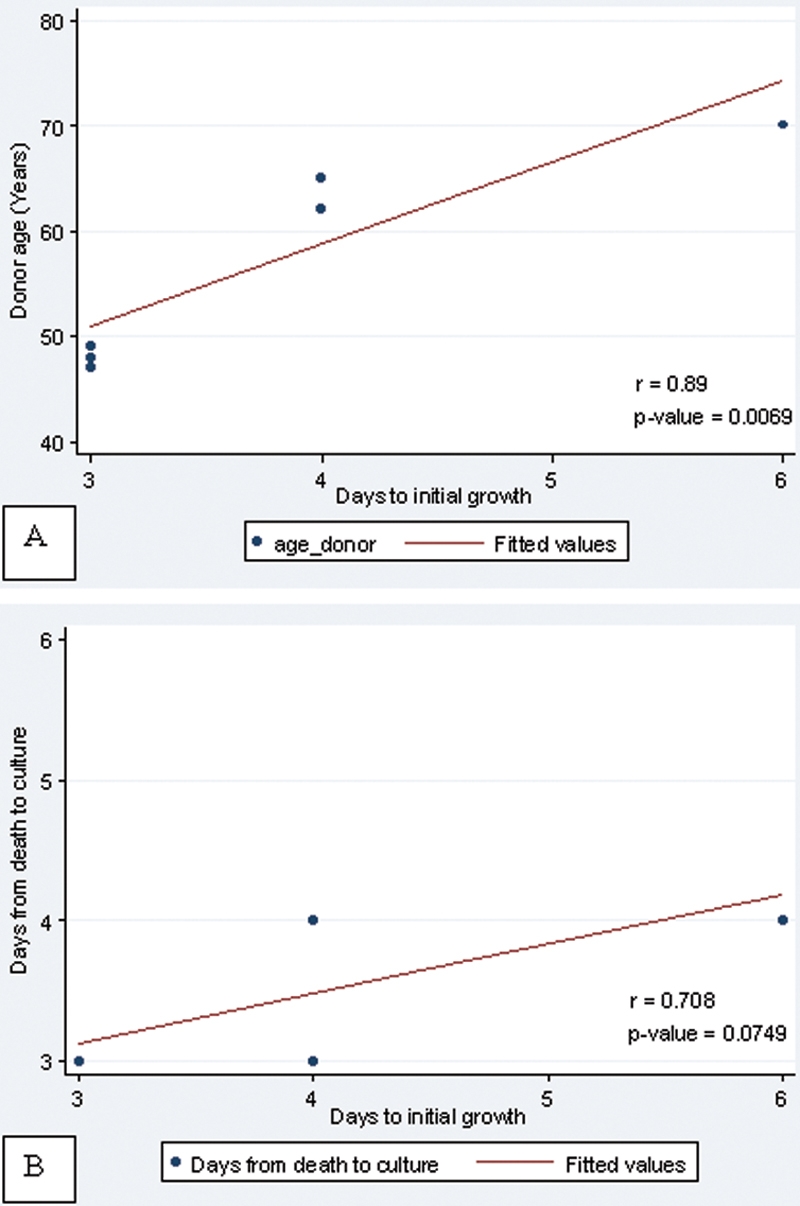
Relationship between the donor ages, time from death to culture and time to initial growth in cultivated corneal epithelial stem cells. **A**: Correlation graph between the donor ages versus time to initial growth. Cultured cells from younger donors significantly grew faster than cells from older donors (p=0.0069). **B**: Correlation graph between the time from death to culture versus time to initial growth. The shorter the time from death to culture, the faster initial cell growth was observed, although not significantly (p=0.0749).

A total 10 of corneoscleral tissues from donors in the age range of 47−70 years were obtained from the Thai Red Cross Eye Bank (average age=57.57 years, standard deviation [SD]=9.36 years). These tissues were harvested and preserved within 24 h after death. The time from death to culture was 4.2±1.4 days (range, 3–7 days). The percentage of corneas from which successful cultures were established was 70%. Epithelial cells from the explants mostly began to migrate onto acellular HAM, forming a rim around the limbal fragment within six days (average 3.86±1.07 days, range 3–6 days). The monolayer to double layers of cells then slowly expanded to cover the HAM until day 21 when the outgrowths were processed. Successful cell growth appeared to depend on the tissue freshness. The cell expansion seemed to be faster from tissue obtained from young donors compared to older donors (p<0.05). Also, there was a trend toward faster initial cell growth for tissues with shorter time from death to culture, which, however, failed to reach statistical significance (p=0.07; Figure 1). At three weeks of culture, limbal corneal epithelial cells covered approximately 50% of the HAM area, and fibroblast-like cells started to be observed.

**Figure 2:**
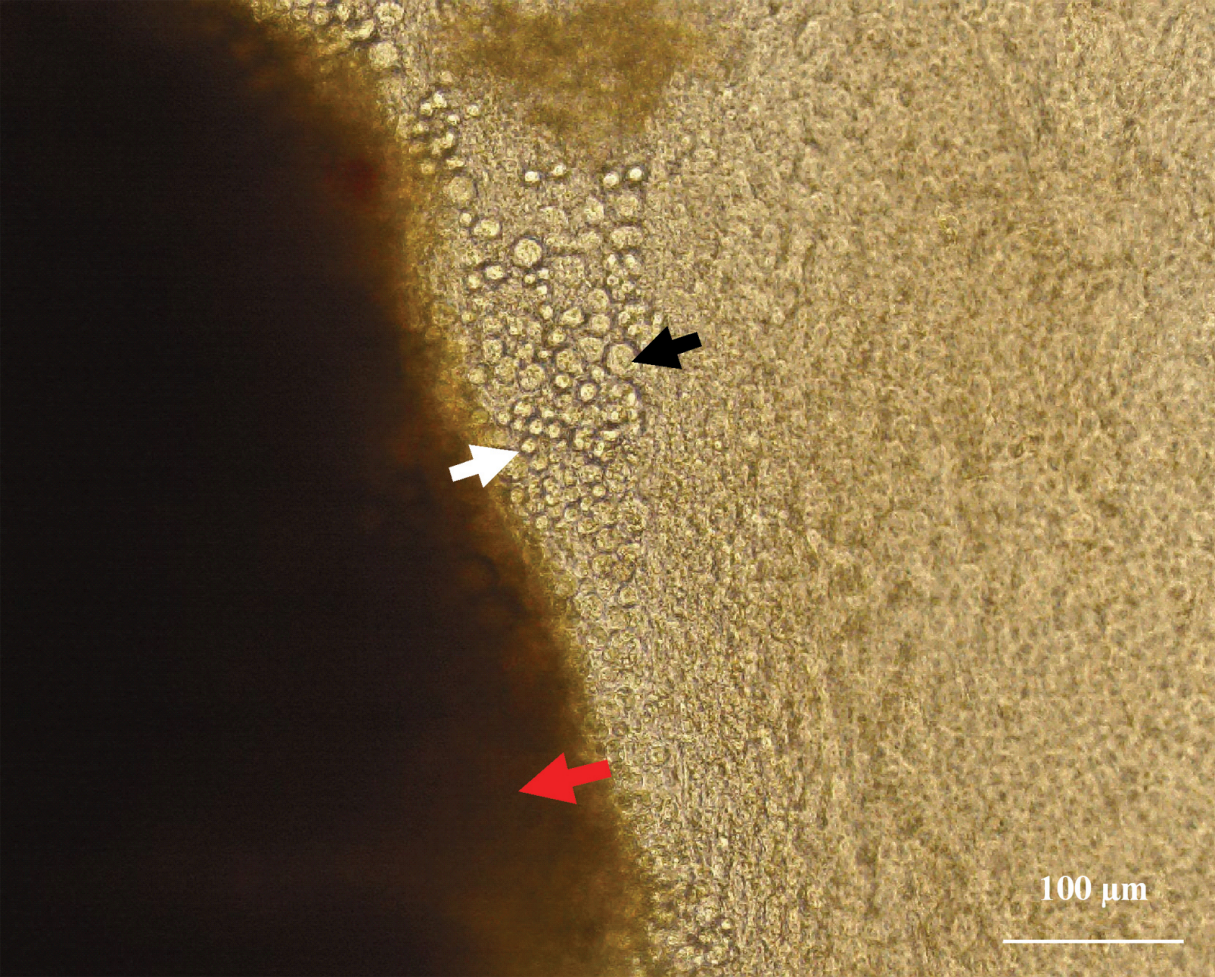
Demonstration of limbal cultures as observed under phase contrast microscope. The corneal epithelial cells were proliferating from the periphery of the explant (red arrow) onto the denuded human amniotic membrane in the absence of feeder cells and serum. The cells adjacent to the explant appeared to be smaller and more uniform and had large nuclei (white arrow) compared to the cells that expanded further away from the explant (black arrow). Magnification: 200X.

Cultured epithelial cells were assessed under the phase contrast microscopy. The cells exhibited a cobblestone like morphology with different size, shape, and nuclei/cytoplasm ratio. The cells adjacent to the explant were smaller and more uniform and had large nuclei whereas the cells further from the explant had a variable cell size and shape with low nuclei/cytoplasm ratio (Figure 2).

### Marker expression

The phenotypic evaluation of the corneal epithelial cultures was performed by RT-PCR and immunocytochemical staining for their expression of putative stem cell markers including nuclear protein p63, ABCG2, K15, and K19 and differentiation markers such as K3, K12, connexin 43, and p75.

### RT-PCR analysis

**Figure 3:**
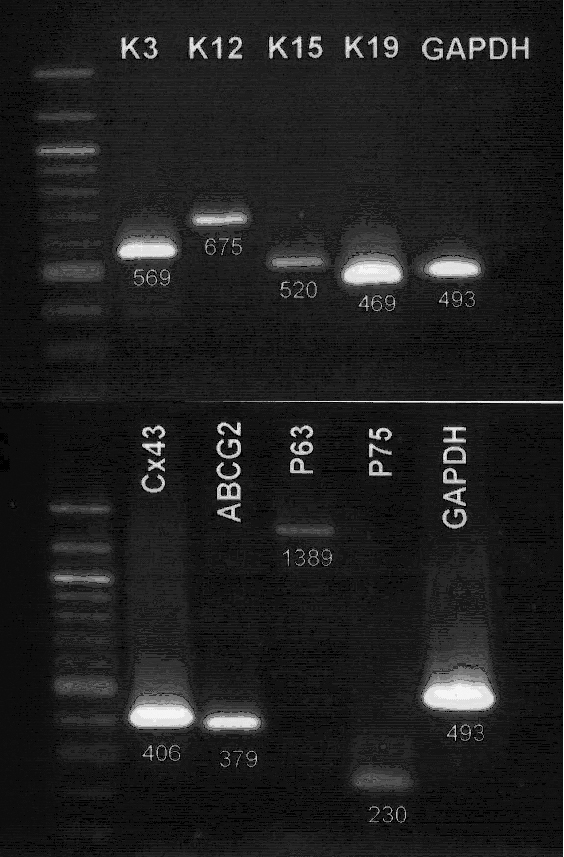
RT-PCR for putative LSC markers and differentiation associated markers. *K3*, *K12*, *K15*, *K19*, *connexin 43*, *p63*, *p75*, and *ABCG2* were all expressed by the cultured corneal epithelial cells. *GAPDH*, a housekeeping gene, was used as an internal control.

With the house keeping gene, *GAPDH*, as an internal control, RT-PCR disclosed an expression of both putative LSC markers (*p63*, *ABCG2*, *K15*, *K19*) and differentiation-associated markers (*K3*, *K12*, *connexin 43*, *p75*) in the cultured cells (Figure 3).

### Immunocytochemical staining

**Figure 4:**
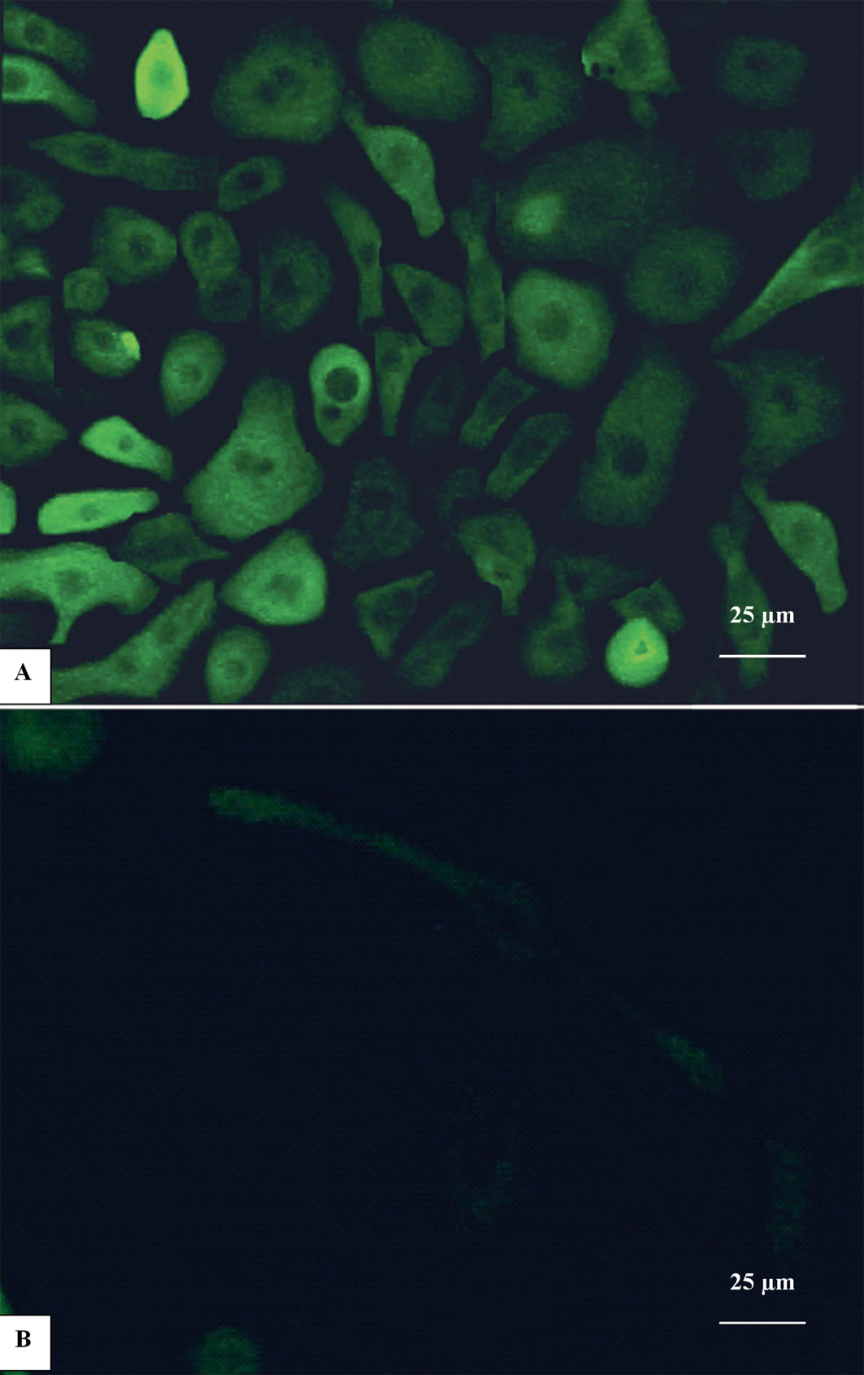
Immunocytochemical staining of human corneal epithelial culture from limbal explants. **A**: Cultured corneal epithelial cells expressed cytokeratin 3 (K3) which was a marker of differentiated corneal epithelium. Positive K3 staining was confined to the cytoplasm. **B**: There was no staining in negative control. Magnification: 400X.

**Figure 5:**
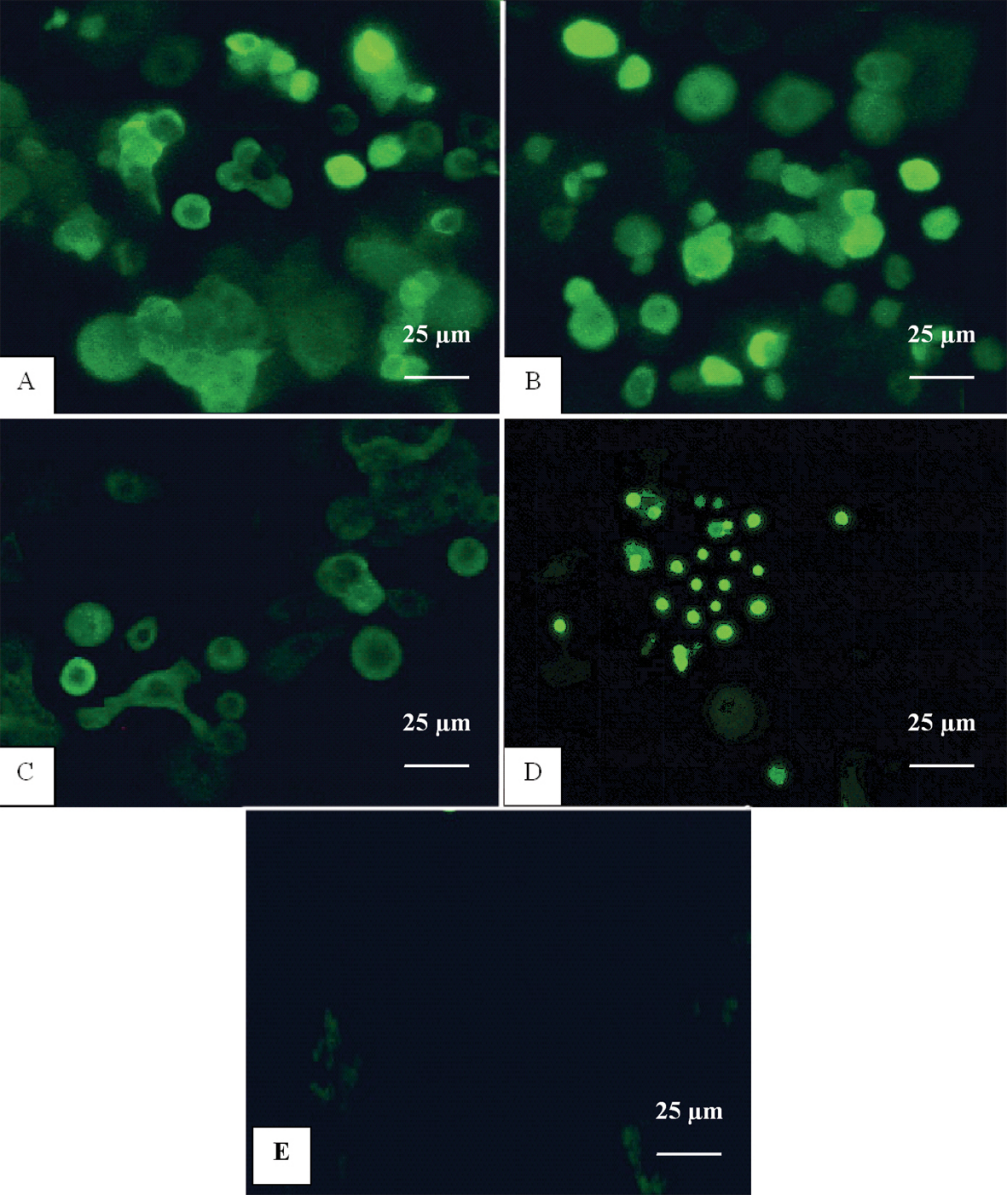
Immunofluorescent staining of human corneal epithelial culture from limbal explants. **A**: Staining of K15 in cytoplasm of cultured corneal epithelial cells was observed. **B**: Cells also showed immunoreactivity for K19 in cytoplasm. **C**: Expression of ABCG2 in the cell membrane and cytoplasm was seen. **D**: Some cells revealed positive staining for p63 in nucleus. **E**: No staining was observed with negative control. Magnification: 400X.

**Figure 6:**
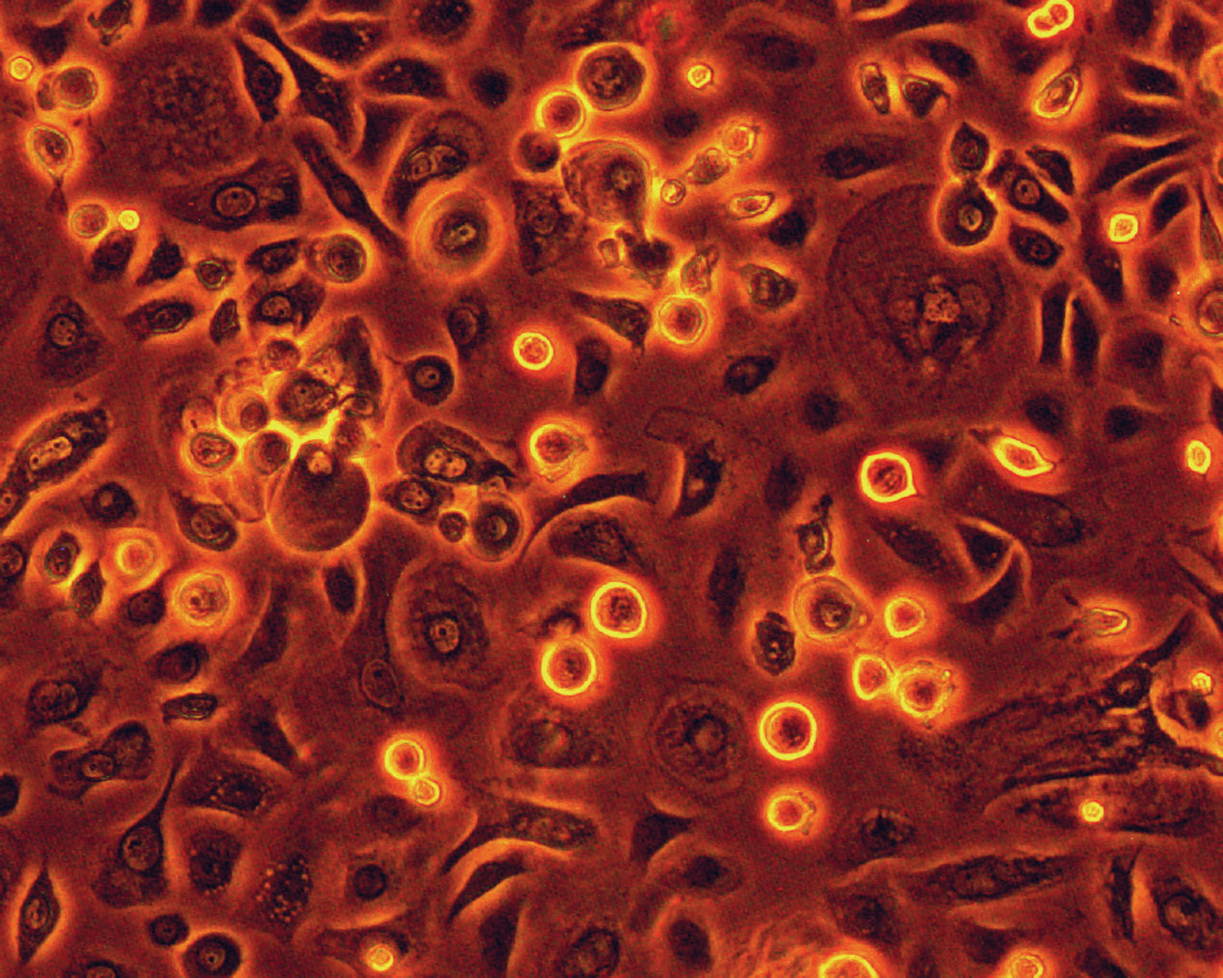
Expression of ABCG2 on cultured corneal epithelial cells. ABCG2, the putative LSC marker, was expressed on the smaller and more uniform cells.

Strong staining of K3 and K12 was present throughout the cultures, indicating the corneal phenotype of the cultured cells. The positively stained cells were estimated to occupy about 50-90% of the cultured cells (Figure 4). However, populations of negative cells were still present. With greater magnification, the positively stained cells appeared larger and more irregular in shape compared to negatively stained cells. Similarly, the cultured epithelial cells were immunopositive for connexin 43 and p75 in the corresponding pattern. Meanwhile, K15 and K19 showed a scattered positive staining throughout the cell sheet (approximately less than 50%). There were also a few ABCG2 positive cells present in a patchy distribution over the cell sheet. Although the cultured cells were stained with nuclear protein p63, the p63 positive staining was generally weaker than other markers (Figure 5). In addition, we found that most of the cultured corneal epithelial cells immunopositive for putative LSC markers were noticeably smaller and more uniform than cells positively stained with differentiation-associated markers (Figure 6). More importantly, the expression of the putative positive stem cell markers seemed to decrease toward the periphery of the outgrowth while the differentiation-associated cell markers were increasingly expressed away from the explant.

Overall, the immunostaining pattern revealed that the putative LSC markers were strongly expressed by small corneal epithelial cells grown nearby the limbal explants. In contrast, larger cells further away from the explant were strongly stained with differentiation-associated cell markers.

## Discussion

Nowadays, several different techniques for limbal epithelial cell cultivation have been published in the literature for which the investigational protocols used considerably involved one or more animal-derived products such as murine 3T3 feeder layer and fetal bovine serum (FBS) [41]. Therefore, co-culture of human limbal stem cells with animal cells or FBS raises concern about infection with recognized or unknown agents [42]. The known potential risks of the murine 3T3 feeder layer include xenogenic microchimerism, xenoantigenicity, and disease transmission through contamination with viruses or prion agents [43,44]. Bovine products such as FBS or bovine pituitary extracts also have a variable likelihood of transmissible spongiform encephalopathies [45]. Although there was a very low probability of potential harm in regard to the risks, a significant risk of disability or death could develop if such an event occurs [41]. Furthermore, these consequences may be more likely in patients receiving allogeneic grafts combined with immunosuppression. For that reason, it would be preferable to culture cells for human transplantation under xenobiotic-free conditions that can maintain stem cells. There have been several reports of successful cultivation of human corneal epithelial stem cells by using media containing autologous serum instead of FBS [46-48]. Nonetheless, the use of autologous serum may be contraindicated in particular patients such as those with significant cerebrovascular or cardiovascular disease, anemia, active bacterial or fungal infection, and positive viral serology (for hepatitis B virus, hepatitis C virus, and human immunodeficiency virus). Additionally, there is no guideline on the use of allogeneic serum for cultivation of allogeneic tissues, and there is still a controversy whether the donor’s or recipient’s sera should be used in such cases. Recently, human corneal epithelial cells have been successful grown in cultures using Epilife® media without serum and feeder cells [49]. However, the phenotype of the corneal epithelial equivalent, including mRNA expression of molecular markers and immunohistological findings have yet been reported. Thus, this experiment was conducted using Epilife® basal medium with a growth supplement and an explant technique without 3T3 feeder cells. Though we could not absolutely eliminate the possibility of contamination by animal-derived factors because the growth supplement still contained purified BSA and purified bovine transferrin, we could at least lower the risk of potential disease transmission.

The cultured cell sheets obtained by this technique had a monolayer to double layer of cells with cobblestone-like morphology. The lack of the stratification of the epithelial cell cultures was probably partly due to the submerged conditions without the air-lifting technique. Another possible explanation was the differences in composition between the Epilife® basal medium and DMEM/F12 medium [49], which has been conventionally used in corneal epithelial stem cell cultivation. The differences between the two media may result in the differences in cellular behaviors. Additionally, the cells close to the limbal explant were smaller and more uniform and had large nuclei. Conversely, the cells further from the explant had a variable cell size and shape with low nuclei/cytoplasm ratio. Immunocytochemistry also revealed that the cells adjacent to the limbal explant appeared to have a higher expression of the putative positive LSC markers. On the other hand, the differentiation-associated markers were poorly expressed close to the explant but showed increased expression away from the explant. This may suggest that there were likely differentiation changes as cells migrated away from the explants similar to findings in a previous report [50].

In addition, the growth of cells in this study seemed to be slower compared to those reported in other studies. The clusters of corneal epithelial cells were seen at the edge of the explant within three days and reached confluence, covering the entire HAM within a period of two to three weeks in the previous studies. Meanwhile, in this study, early small epithelial cell colonies were observed within six days and reached confluence, covering only 50% of the HAM area in three weeks. [35,46,51]. These findings plus the presence of fibroblast-like cells in the cultures at three weeks may also be caused by the different media and culturing system used in this study, which might influence the cellular response.

Although there was a seemingly delayed onset of cell expansion and no stratified growth of cells on HAMs, the cells still expressed cytokeratin 3 and 12, which are considered markers of corneal differentiation. Also, limbal corneal epithelial cells cultured in the serum- and feeder-free Epilife® media exhibited other features of differentiated corneal epithelial cells as suggested by the positive staining for p75 and connexin. This observation is similar to those cultured in the conventional medium consisting of FBS and feeder cells. Furthermore, some cells showed positive staining for proposed limbal stem cell markers (K15, K19, ABCG2, and p63), indicating that these cells were still able to maintain the corneal limbal stem cell properties with this culture technique. This culture system also obviated the need for FBS and use of culture inserts. Given safety and feasibility considerations, this technique may offer a reasonable way to expand corneal epithelium in culture. However, growing cells on different substrates or a modifying stem cell culture environment should be further investigated to find a LSC niche that can maintain “stemness” and prevent stem cell differentiation. Moreover, studies regarding the components of Epilife® media and its cellular response as well as the metabolic activity and other stem cell properties of cell cultures have yet to be determined. With further extensive improvement, this technique may become an alternative to the conventional culturing method in the future.

In conclusion, our study demonstrates that human limbal corneal epithelial cells can be cultivated using a simple explant technique with Epilife® culture medium under serum- and feeder-free condition. This culture system may also be useful for the clinical application of limbal stem cell culture as well as the further investigations of stem cell characteristics.
